# ‘Tablet-in-Syringe’: A Novel Dosing Mechanism for Dysphagic Patients Containing Fast-Disintegrating Tablets Fabricated Using Semisolid Extrusion 3D Printing

**DOI:** 10.3390/pharmaceutics14020443

**Published:** 2022-02-18

**Authors:** Pattaraporn Panraksa, Bin Zhang, Pornchai Rachtanapun, Kittisak Jantanasakulwong, Sheng Qi, Pensak Jantrawut

**Affiliations:** 1 Department of Pharmaceutical Sciences, Faculty of Pharmacy, Chiang Mai University, Chiang Mai 50200, Thailand; pattaraporn.prs@gmail.com; 2 School of Pharmacy, University of East Anglia, Norwich NR4 7TJ, UK; b.zhang2@uea.ac.uk; 3 Division of Packaging Technology, Faculty of Agro-Industry, School of Agro-Industry, Chiang Mai University, Chiang Mai 50100, Thailand; pornchai.r@cmu.ac.th (P.R.); jantanasakulwong.k@gmail.com (K.J.); 4 Cluster of Agro Bio-Circular-Green Industry (Agro BCG), Chiang Mai University, Chiang Mai 50100, Thailand

**Keywords:** 3D printing, extrusion-based 3D printing, semisolid extrusion 3D printing, dysphagia, fast-disintegrating tablets, phenytoin sodium

## Abstract

With the ability to fabricate personalized dosage forms and considerably shorter manufacturing time, semisolid extrusion (SSE) 3D printing has rapidly grown in popularity in recent years as a novel, versatile manufacturing method that powers a wide range of applications in the pharmaceutical field. In this work, the feasibility of using SSE 3D printing to fabricate fast-disintegrating tablets (FDTs) that are pre-filled in dosing syringes was evaluated. The novel design approach, ‘tablet-in-syringe’, was aimed to ease the oral drug administration and improve the dosing accuracy for dysphagic patients. The effect of varying polymer (hydroxypropyl methylcellulose E15) concentrations and printing parameters (e.g., extrusion rate) on dimensional accuracy, physicochemical properties, disintegration time, and content uniformity of 3D-printed FDTs was studied. An overall comparison of results demonstrated that the best FDT formulation among those developed was with a polymer:drug ratio (*w*/*w*) of 1:30, printed at extrusion rate of 3.5 μL/s. The diameter of printed filaments of this formulation was observed to be similar to the nozzle diameter (22G), proving that good printing accuracy was achieved. This FDTs also had the fastest disintegration time (0.81 ± 0.14 min) and a drug (phenytoin sodium, as the model drug) content uniformity that met pharmacopeial specifications. Although the flow characteristics of the dissolved formulation still need improvement, our findings suggested that the novel ‘tablet-in-syringe’ could potentially be considered as a promising fast-disintegrating drug delivery system that can be personalized and manufactured at—or close to—the point of care for dysphagic patients using SSE.

## 1. Introduction

Oropharyngeal dysphagia (OD), also known as swallowing difficulties, is a symptom of swallowing dysfunction that provokes difficulty or inability to safely propel a food bolus from the mouth, through the pharynx, to the esophagus [1]. OD is a growing global healthcare concern associated with a wide range of diseases and health conditions, including neurological or neurodegenerative diseases (e.g., Parkinson’s disease, Alzheimer’s disease, stroke, dementia, multiple sclerosis (MS), traumatic brain injury) and head and/or neck diseases (e.g., head and neck cancer, osteophytes) [2,3,4]. It is an increasingly common symptom that can occur at any age with an estimated prevalence of 8% of the general population [5], but is most commonly diagnosed in the elderly, aged 65 and older, with occurrence rates of up to 30–40% in the population aged ≥ 65 years old [6]. The prevalence of OD in the elderly is also expected to substantially increase in the coming years as the elderly population continues to expand. Hence, it becomes the challenge of researchers and healthcare professionals to find the most effective and safest way possible to manage this difficulty in order to avoid the risk of negative health status changes caused by dysphagia, such as the risk of malnutrition and pneumonia, and to improve the patients’ quality of life, because accurate swallowing is a necessary physiological function for safe breathing and alimentation [7].

The traditional approach to facilitating swallowing is to modify the consistency of the liquid dosed to patient and match the texture to the patients’ swallowing abilities [8]. In order to effectively deliver oral medications to dysphagic patients, one of the common practices is to mix the crushed tablets or opened capsule fillings with thick liquid (unlicensed administration) to adjust the viscosity and flow characteristics of the drug solutions to ensure that the formulation would be suitable for the dysphagic patient to swallow [9,10]. According to the International Dysphagia Diet Standardization Initiative (IDDSI) framework [11], with regard to liquids, the IDDSI committee classified drink thickness into 5 levels (from 0 to 4) based on fluidity and scientific and survey evidence, with level 0 being a thin liquid with a water-like flow. Level 1 is a slightly thick liquid that is frequently used as a thickened drink in the pediatric population or the adult population when swallowing safety must be controlled. Level 2 is a mildly thick liquid that is appropriate for patients who have lost tongue control and strength. Level 3 is a liquidized/moderately thick liquid that is better suited for patients who have difficulty swallowing or have pain on swallowing. IDDSI levels 2–4 are considered appropriate for adult oropharyngeal dysphagia management. However, for IDDSI level 4, which describes extremely thick liquids that cannot be passed through a 10 mL syringe in 10 s, the additional measurements should be evaluated using IDDSI food testing methods (fork test and spoon tilt test).

**Figure 1:**
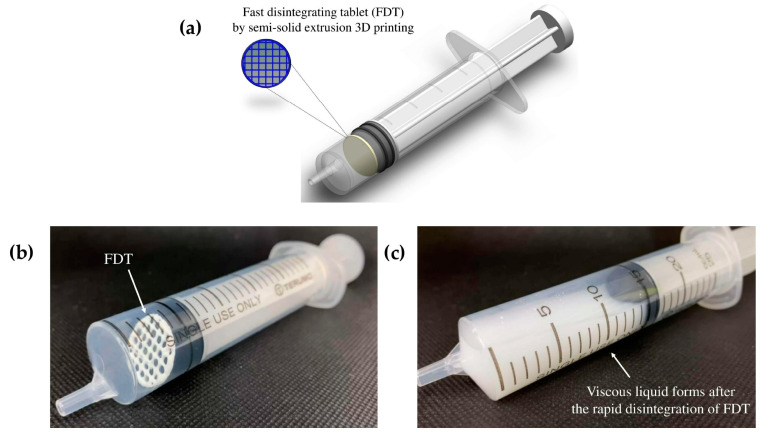
Graphical illustration of the ‘tablet-in-syringe’ device (**a**) and images of 3D-printed fast-disintegrating tablet (FDT) pre-filled in a dosing syringe (**b**), and after disintegration (**c**).

Using such unlicensed administration approaches often increases the risks of altering the bioavailability of the original solid dosage form due to the potential interaction with food, and could potentially put the patient in danger of dose dumping, if the solid dosage form is intended to be a controlled-release product. This study proposes a new oral dosing device for patients with dysphagia to improve the dosing accuracy without the need of tempering. ‘Tablet-in-syringe’ is a dosing device where a fast-disintegrating tablet (FDT) is 3D printed and pre-filled in a dosing syringe, as illustrated in Figure 1. A fixed amount of water can be drawn into the syringe to disintegrate the tablet rapidly. The syringe can then be used to directly dose the patient orally. However, the disintegrated FDT formulation needs to provide sufficient thickness that is suitable for administrating to dysphagia patients, according to IDDSI guidance.

Fast-disintegrating tablets (FDT) are one of the promising dosage forms that can rapidly disintegrate in the mouth or rapidly disintegrate in water before being administered orally via syringe. The disintegrated mass of the FDT could then be gradually moved down and passed through the esophagus, allowing pediatric, geriatric, psychiatric, bedridden, and dysphagic patients to take their medications with ease [12,13]. Furthermore, FDT offers several advantages, such as high drug loading, good chemical stability, rapid onset of action, improved bioavailability, and no need to measure drug dosing (single-unit dosage forms) [14]. For the development and manufacture of FDT, the porosity, density, and hardness are some of the FDT properties that must be considered during the development process. In general, the FDT should have the highly porous network, low density, and low hardness to promote fast disintegration [15]. To date, various manufacturing techniques have been adopted to fabricate FDTs, such as granulation methods [16], freeze drying [17], sublimation [18], direct compression [19], and three-dimensional (3D) printing technology [20,21]. Among these techniques, 3D printing technology is noteworthy regarding its flexible and digitally controllable design and manufacturing process, which enables the design and development of the desired porous and loose structure of FDT, thereby accelerating disintegration time and reducing swallowing difficulties [22].

Extrusion-based 3D printing is the most common 3D printing method used for pharmaceutical purposes and its potential for fabricating solid oral dosage forms has been extensively researched in recent years [23,24]. Semisolid extrusion (SSE) 3D printing is a subcategory of extrusion-based 3D printing. During an SSE 3D-printing process, the formulated paste or gel (often referred as ‘ink’) is extruded from the printing nozzle and deposited in a layer-by-layer manner to form a 3D object [25]. It is regarded as a very promising approach for the fabrication of various personalized pharmaceutical products, such as polypills, controlled-release tablets, chewable printlets, immediate-release tablets, and fast-disintegrating drug delivery systems (fast-disintegrating films or tablets), that can be tailored to each patient’s clinical need [26]. Although the throughput of 3D printing in comparison with other, traditional, large-scale manufacturing methods is much lower—which limits its production in large-scale manufacturing—3D printing nonetheless remains superior in its ability to produce on-demand, individualized dosage forms on a small scale at, or close to, the point of care [27,28]. However, studies on the feasibility of 3D printing in pharmaceutical applications are still limited and understudied. Only a few studies have attempted to fabricate FDTs through semisolid extrusion 3D printers [29,30,31]. Both the choice of excipients and infill density of the design of the tablets can affect the disintegration time of the FDTs [32].

The aim of this study was to evaluate the feasibility of using SSE 3D printing to produce FDTs with high drug loading of water-soluble drugs. Phenytoin sodium, as one of the most commonly used antiepileptic drugs, was chosen to be the model drug in this study. It is used to treat and control the generalized tonic–clonic (grand mal) and complex partial (psychomotor, temporal lobe) seizures and has a narrow therapeutic index. Therefore, precise therapeutic dosages and dosage adjustments based on the patient’s individual characteristics and plasma concentration [33] are vital for this drug, but are currently not achieved by the commercial drug products. In our previous work [34], we designed, and 3D printed the phenytoin-loaded, orodispersible films (ODFs) using a customized syringe extrusion 3D printer. Our developed ODFs showed promising results in terms of film appearance and mechanical strength as well as a rapid disintegration time of less than 5 s. In this study, we examined the suitability of using SSE 3D printing to print FDTs in order to increase the drug loading, and proposed the new design of the dosing solution for dysphagic patients. The printing inks were formulated as pastes using hydroxypropyl methylcellulose (HPMC E15), a low-viscosity grade, water-soluble polymer, with moderate hydroxypropyl substitution (8.6%) and high methoxy content (28.4%). The effect of printing ink rheology and extrusion rate on printability dimensional accuracy, physical and morphological properties, in vitro disintegration time, phenytoin content, and the International Dysphagia Diet Standardization Initiative (IDDSI) flow characteristics of developed formulations were evaluated. 

## 2. Materials and Methods

### 2.1. Materials

The model drug, 5,5-diphenylhydantoin sodium salt or phenytoin sodium salt (PT), with purity of ≥99% was purchased from Sigma-Aldrich (Saint Louis, MO, USA). Hydroxypropyl methylcellulose E15 (HPMC E15, AnyCoat^®^-C AN15, substitution type 2910, viscosity 15 mPa·s) was purchased from Lotte Fine Chemical Co., Ltd. (Seoul, Korea). Sodium starch glycolate (Glycolys^®^) was purchased from Roquette (Lestrem, France). Ethanol (VWR Chemicals BDH^®^, Radnor, PA, USA) and distilled water were used as the solvent for preparing the printing ink formulations. All of the other reagents and solvents used in this study were analytical grade.

### 2.2. Preparation of Printing Inks

The drug-loaded printing inks were prepared by dispersing phenytoin sodium at a concentration of 1.05 g/mL in ethanol–water mixtures (9:1 *v*/*v*). The drug dispersion was magnetically stirred for 2 h at 400 rpm and 60 °C, followed by the addition of sodium starch glycolate (SSG) as a superdisintegrant at a concentration of 4% *w*/*v* of total formulation, and then stirred for another 30 min. Subsequently, hydroxypropyl methylcellulose E15 (HPMC E15) at the polymer:drug weight ratios (*w*/*w*) of 1:25, 1:30, and 1:35 was added and mixed at room temperature with a spatula until the homogeneous semisolid system of printing inks was formed. Afterwards, the printing inks were kept in tightly sealed and light-protected beakers at room temperature for a day before 3D printing.

### 2.3. Rheological Characterisation of Printing Inks

The rheological characteristics of all printing inks were characterized by the Brookfield Rheometer (R/S-CPS, P25 DIN plate, Brookfield engineering laboratories, Middleboro, MA, USA) equipped with 25 mm in diameter of parallel plates, set at a gap width of 1 mm, and operated in controlled shear rate (CSR) mode. For all tests, approximately 0.6 mL of each printing ink sample was gently loaded onto the lower plate geometry and the excess printing ink sample was carefully removed to suit the 25 mm plate diameter. The shear viscosity tests were carried out in flow ramp mode, with the shear rate gradually increasing from 0 to 100 s^−1^ in 1 min, and the temperature was controlled at 25 °C. All the tests were carried out in triplicate. The rheology of all printing inks was analyzed and the flow behavior or power-law index (n) and consistency coefficient (K) were calculated using the power-law model equation, as follows:

$$\eta =K{\dot{\gamma }}^{n-1}$$

where η is the viscosity of the printing ink measured in Pa·s, K is the consistency coefficient (Pa·s^n^), $\dot{\gamma }$ is the shear rate measured in s^−1^, and n is the power-law index.

### 2.4. Design and SSE 3D Printing of FDTs

**Figure 2:**
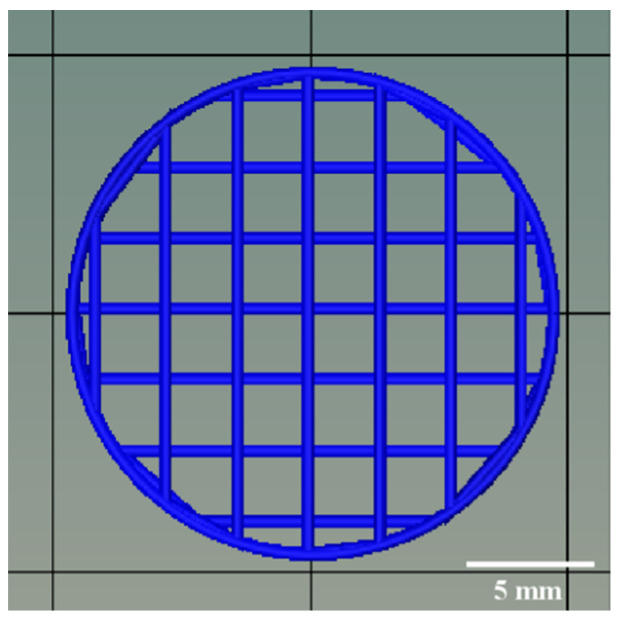
Computer-aided design (CAD) model of the 3D-printed FDT.

The model of 3D-printed FDTs in cylindrical shape was predesigned and created using the computer-aided design (CAD) software and then exported in the stereolithography (STL) file format. As shown in Figure 2, the diameter and thickness of the 3D model were designed to be 19.0 mm and 1.0 mm, respectively. In addition, based on preliminary optimization results (data not shown), the 3D-printed FDTs were designed to have a porous grid structure with a 25% infill density and a layer height of 0.41 mm, which is equivalent to the inner diameter of a 22G nozzle. Subsequently, the printing inks were transferred into a 3 mL syringe (Terumo, Tokyo, Japan) and printed with an SSE 3D printer (BIOX 3D printer, Cellink, Boston, MA, USA). The stepper motors drive the motion in the Z direction via twin lead screws with an overall resolution of 0.001 mm/step with a 1.8° step angle. During the printing process, the nozzle speed was kept at 10 mm/s. The extrusion rate was varied to 3.0, 3.5, and 4.0 μL/s, which corresponded to an estimated printing time of 0.8–1.2 min per tablet, to investigate its effect on the dimensional and pore geometry accuracy of the 3D-printed FDTs. After printing, the 3D-printed FDTs were dried at room temperature for 24 h to remove solvents.

### 2.5. Dimensional Accuracy and Filament Fusion Analysis

To evaluate the printing accuracy and shape stability of the 3D-printed FDTs, the diameter of printing ink filaments extruded through an extrusion nozzle (22G, 0.41 mm in internal diameter) and 2 different factors—shape fidelity (SFF) and rate of material spreading (Df_r_)—were evaluated using Equations (1) and (2), respectively. The diameter and pore area used for calculation were measured in ImageJ (Bethesda, MD, USA) using top view images from a digital camera and scanning electron microscopy (SEM).

SFF = Printed area/CAD model area,(1)

Df_r_ = [(A_t_ − A_a_)/A_t_] × 100%, (2)

where A_t_ is theoretical pore area and A_a_ is actual pore area.

### 2.6. Weight and Thickness Variation of SSE 3D-Printed FDTs

To assess the uniformity of 3D-printed FDTs, ten tablets of each formulation were randomly selected and weighed individually with an analytical weighing balance (LAB 214i, Adam Equipment Co., Ltd., Jing An, Shanghai, China) and measured for their thickness at three different points on a single 3D-printed FDTs using an electronic digital thickness gauge (Deqing Syntek Electronic Technology Co., Ltd., Zhejiang, China). The average weight and average thickness were calculated, along with standard deviation (SD).

### 2.7. Morphological Assessment of SSE 3D-Printed FDTs

Scanning electron microscopy (SEM) images of 3D-printed FDTs were acquired using JEOL JCM-7000 NeoScope™ Benchtop SEM (JEOL, Tokyo, Japan). Prior to imaging, uncoated 3D-printed FDTs were mounted on aluminum stubs using double-sided carbon tape (NEM tape, Nisshin Co., Ltd., Tokyo, Japan), followed by gold-coating for 2 min, then positioned on the stage in the imaging compartment of the device. Then, SEM images of all the 3D-printed FDTs were collected using a SE (secondary electron) detector at an acceleration voltage of 5 kV under low vacuum mode. Subsequently, 2D assessment of 3D-printed FDTs morphology, pore interconnectivity, and pore geometry was conducted at magnifications of ×30.

### 2.8. In Vitro Disintegration Time Tests of SSE 3D-Printed FDTs

The disintegration time of the 3D-printed FDTs for oral administration via syringe was determined by placing the tablet into the barrel of a 20 mL syringe (Terumo, Tokyo, Japan) and adding 5 mL of air. Ten milliliters of 37 °C water was then drawn into the syringe and gently shaken manually by simple downward–upward inversion of the syringe. The time required for the 3D-printed FDTs to break into small pieces was visually recorded and noted as in vitro disintegration time.

### 2.9. Determination of Phenytoin Sodium Content Uniformity

To determine the phenytoin sodium content in the 3D-printed FDTs, 3 tablets of each formulation were taken in separate 25 mL vial, 10 mL of distilled water was added and continuously magnetically stirred at a speed of 500 rpm at room temperature for 2 h. Then, the sample solution was suitably diluted 3.75 times with methanol and further diluted 8 times with distilled water prior to filtering through a 0.45 µm nylon membrane filter (Alwsci^®^ Technologies, Shaoxing, China) and analyzed by high-performance liquid chromatography (HPLC). The quantitative analysis of phenytoin sodium was performed using an HPLC system (HP 1100 Series HPLC, Agilent Technologies, Inc., Santa Clara, CA, USA) equipped with a C18 column (Capcell Pak AQ 250 mm × 4.6 mm, particle size of 5 µm, Shiseido, Tokyo, Japan) and the analysis method was adopted from the United States Pharmacopeia (USP: extended phenytoin sodium capsules) [35]. The HPLC analysis was carried out at 25 °C using an isocratic mobile phase of methanol–water (70:30, *v*/*v*). The filtered mobile phase was pumped at a flow rate of 1.0 mL/min with run time of 8.0 min. The injection volume was 10 µL and UV detection was carried out at 229 nm with a retention time of approximately 4.5 min. The phenytoin sodium contents were calculated using a standard calibration curve for phenytoin sodium in water, which was constructed in the range of 0.10–0.60 mg/mL and demonstrated linearity with a high correlation coefficient (r^2^ = 0.9992). The linear regression equation was obtained as *y* = 9463.3*x* − 482.16, where *y* and *x* correspond to peak area and phenytoin sodium concentration (mg/mL), respectively. The limits of detection (LOD) and limits of quantification (LOQ) were determined as 0.20 and 0.61 µg/mL, respectively. All the measurements were performed in triplicate and the average percentages of phenytoin sodium content were calculated with the standard deviation. The optimum formulation in terms of the dimensional accuracy, disintegration time, and phenytoin sodium content uniformity were selected for further study on its mechanical property, in vitro release profile, release kinetics, and IDDSI flow characteristics.

### 2.10. Mechanical Strength Testing of SSE 3D-Printed FDTs

The mechanical strength testing of the 3D-printed FDT was adapted from the study of Zhao et al. [36]. The test was performed by using a texture analyzer (TX.TA plus, Stable Micro Systems, Surrey, UK) equipped with a 5 kg load cell, a 2 mm stainless steel cylindrical probe (P/2 probe) at temperature of 25 °C. Prior to the test, the diameter and thickness of each 3D-printed FDT were measured by using an electronic digital thickness gauge. The test was conducted in compression mode with a pre-test speed of 1 mm/s, a test speed of 0.1 mm/s up to a distance of 2 mm, a post-test speed of 1 mm/s, and a trigger force of 5 g. The maximum force reading was noted as hardness of the 3D-printed FDTs [37], whereas the tensile strength of the 3D-printed FDTs was characterized by the maximum breaking force, and the diameter and thickness of the 3D printed FDTs were calculated from the following equation [36]:

$$\sigma =\frac{2F}{\pi DH}$$

where σ is the tensile strength (TS; N/mm^2^), F is maximum breaking force (N), D is the probe diameter (mm), and H is the thickness of 3D-printed FDT.

All measurements were carried out in five replicates and the hardness and tensile strength of the selected 3D-printed FDT were reported as mean ± standard deviation (SD).

### 2.11. In Vitro Phenytoin Sodium Release Study and Drug Release Kinetics

The in vitro release behaviors of the most optimal 3D-printed FDT formulation were investigated using a USP Apparatus 2 (paddle method) modified from a USP monograph on phenytoin oral suspension performance tests [35]. To determine phenytoin sodium release in suspension dosage form, 10 mL of 3D-printed FDT sample suspension (after disintegration) was vigorously shaken about 100 times and its density was determined using a 10 mL pycnometer (Witeg Labortechnik GmbH, Wertheim, Germany). Then, a total of 10 mL of sample suspension was collected using a 10 mL syringe, and the total weight of syringe and sample was recorded. Thereafter, with the paddles lowered, the sample suspension in each syringe was gently emptied into the bottom of each dissolution vessel containing 900 mL of tris(hydroxymethyl)aminomethane with 1% *w*/*v* sodium lauryl sulfate (SLS) buffer solution (pH 7.5). Each syringe was then reweighed and the weight of sample suspension which delivered into each vessel was calculated. The release study was performed in 6 replicates at a paddle speed of 35 rpm and 37 ± 0.5 °C. At predetermined time intervals (1, 3, 5, 10, 15, 30, and 60 min), 3 mL of the sample was withdrawn and replaced with an equal volume of fresh dissolution medium in order to maintain sink conditions throughout the experiment. The withdrawn dissolution samples were filtered with a 0.45 µm nylon membrane filter prior to HPLC analysis. For HPLC analysis, the chromatographic separation was performed at 25 °C on a C18 column with an isocratic mobile phase of 23% *v*/*v* acetonitrile, 27% *v*/*v* methanol and 50% *v*/*v* of pH 3.0 phosphate-buffered solution at a flow rate of 1.0 mL/min. The injection volume was 10 µL and the UV detection wavelength was set as 240 nm. The retention time was approximately 7.8 min. The LOD and LOQ were found to be 0.14 and 0.41 μg/mL, respectively. The cumulative percentage of drug release was calculated using the standard equation from the standard calibration curve of phenytoin sodium in Tris buffer pH 7.5 with 1% *w*/*v* SLS: *y* = 5.2578*x* + 3.419 (r^2^ = 0.9998), where *x* and *y* correspond to phenytoin sodium concentration (µg/mL) and peak area, respectively.

In order to determine the kinetics and mechanism of drug release, various kinetics models (i.e., zero-order model, first-order model, Higuchi matrix model, and Korsmeyer–Peppas empirical power-law model) were applied to the data obtained from in vitro release study. The in vitro release data were fitted into the following equations:

$$(a)\;\text{zero-order}\;\mathrm{model}:\;Q_{t}=Q_{0}+k_{0}\times t$$

where $Q_{t}$ is the amount of drug dissolved in time (t), $Q_{0}$ is the initial amount of drug in the solution, and $k_{0}$ is the zero-order release constant.

$$(b)\;\text{first-order}\;\mathrm{model}:\;\log Q_{0}-\log Q_{t}=\frac{k_{1}\times t}{2.303}$$

where $Q_{0}$ is the initial concentration of the drug, $Q_{t}$ is the amount of drug dissolved in time (*t*), and $k_{1}$ is the first-order release constant.

$$(c)\;\mathrm{Higuchi}\;\mathrm{matrix}\;\mathrm{model}:\;Q_{t}=k_{H}\times t^{1/2}$$

where $Q_{t}$ is the amount of drug dissolved in time (t) and $k_{H}$ is the Higuchi diffusion constant.

$$(d)\;\mathrm{Korsmeyer}–\mathrm{Peppas}\;\mathrm{empirical}\;\text{power-law}\;\mathrm{model}:\;\frac{M_{t}}{M_{\infty }}=k\times t^{n}$$

where $\frac{M_{t}}{M_{\infty }}$ is the fraction of drug released at time (t), k is the structural and geometrical constant, and n is the release exponent.

### 2.12. International Dysphagia Diet Standardisation Initiative Flow Test

In order to determine the swallowing safety of 3D-printed FDTs when administered orally via syringe after disintegration in warm water and/or other liquids, the flow characteristics were measured using the drink testing method described in the International Dysphagia Diet Standardization Initiative (IDDSI) framework and guidelines [38]. In the IDDSI flow test, in accordance with the ISO standard (ISO 7886-1) and IDDSI syringe specifications, a single-use 10 mL central Luer slip tip syringe (REF 302143, BD, Tuas, Singapore) with a 61.5 mm length of 10 mL scale was used in this study. Briefly, 10 mL of each liquid sample was slowly poured into the syringe until it reached the 10 mL mark. Then, the syringe nozzle was released, and the liquid sample was allowed to flow freely for 10 s The remaining volume of the liquid sample in the syringe was determined using video analysis, and an image of the liquid sample was captured after 10 s. The IDDSI level was determined based on the remaining volume of the sample after 10 s of flow as level 3 (more than 8 mL remaining), level 2 (4–8 mL remaining), level 1 (1–4 mL remaining), or level 0 (less than 1 mL remaining).

### 2.13. Statistical Analysis

All data were presented as mean ± standard deviations (SD) and then were analyzed through the one-way analysis of variance (ANOVA) using SPSS^®^ statistics software version 17.0 (IBM Corporation, Armonk, NY, USA) at *p* level less than 0.05 to determine the statistical significance of the difference in the results. 

## 3. Results and Discussion

### 3.1. Rheological Behaviors of Printing Inks

**Figure 3:**
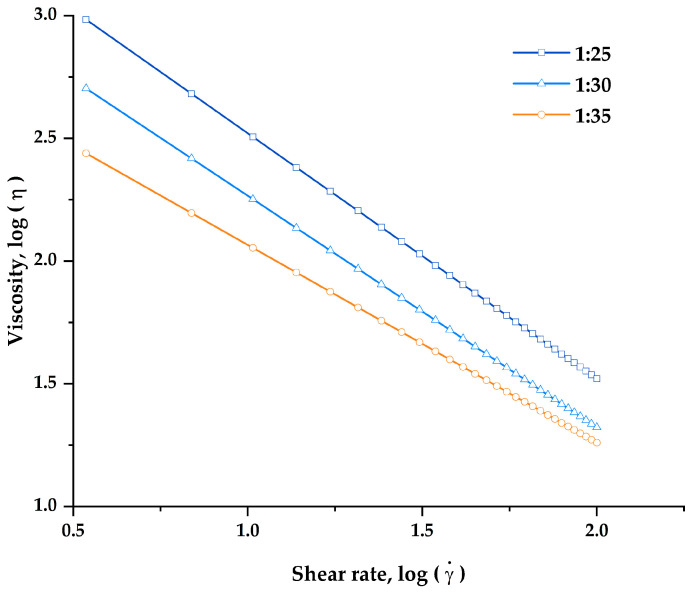
Log–log plot fitting with power-law model of viscosity as a function of shear rate of the printing inks at polymer:drug ratios (*w*/*w*) of 1:25, 1:30, and 1:35.

In this study, the rheological characterization of all developed printing inks was carried out in order to assess the flowability and suitability of printing inks for semisolid extrusion 3D printing. To be suitable for SSE 3D printing, the viscosity of the inks should become less viscous and could be extruded smoothly through the nozzle when the high shear rate was applied. The flow curves (Figure 3) showed that the apparent viscosity of all printing inks was found to decrease significantly as shear rate increased, demonstrating the shear-thinning non-Newtonian fluid properties that make the inks suitable for SSE 3D printing. 

In addition, the power-law model was fitted to experimentally obtained results (viscosity–shear rate flow curves) of all the printing inks to determine both flow behavior or power-law index (n) and consistency coefficient (K), as shown in Table 1. The results showed that the power-law model fits the experimental flow curve well and is appropriate for expressing the rheological behavior of all printing inks, as the correlation coefficient (R^2^) values in all printing inks were greater than 0.99. The n values of all printing inks were found less than 1 and fall in the range of 0.00–0.20, indicating a strong shear-thinning behavior [39]. In addition, the viscosity, n values, and K values of all printing inks were found to be highly dependent on polymer concentration. An increase in the proportion of HPMC E15 resulted in an increase in the K values, indicating that the printing inks became more viscous and more pseudoplasticity at higher HPMC E15 contents [40]. While the n values of all printing inks were found to decrease from 0.19 to 0.00 when the HPMC E15 content was increased and polymer:drug ratio was changed from 1:35 to 1:25, suggesting that the printing ink formulation with a polymer:drug ratio of 1:25 exhibits more intense shear-thinning behavior. This finding is consistent with previous research which reported that the addition of polymer content could significantly affect the flow behaviors of the printing ink by increasing the viscosity and shear-thinning behaviors. The enhanced shear-thinning behaviors of printing inks may also influence their extrusion capability and the structural stability of 3D-printed FDTs after 3D drying [41]. The printable ink should ideally have shear-thinning behavior and a viscosity low enough to allow easy extrusion from a small-diameter nozzle while also being high enough to allow the printing to hold its shape after printing and stackable with previous layers [42].

At the highest HPMC E15 content, printing inks with a polymer:drug ratio of 1:25, which has the highest viscosity at initial shear rate (961.47 ± 81.25 Pa·s), failed to be extruded through continuously the extrusion nozzle (22G, 0.41 mm in diameter). The nozzle blockage of this formulation was observed shortly after the printing began. This could be due to the denser formation of a network structure between the drug and the polymer, as well as excessive viscosity and rapid solvent evaporation. On the other hand, the printing inks with polymer:drug ratios of 1:30 and 1:35 were printable through nozzle diameters of 0.41 mm at extrusion rates of 3.5 and 4.0 μL/s, respectively. According to our findings, a printing ink should have a viscosity in the range of 270–500 Pa·s at an initial shear rate of 3.44 s^−1^ in order to be effectively extruded and 3D printed. Thus, the 1:30 and 1:35 printing ink formulations were subsequently selected for further evaluation for their printing performance and physicochemical properties of the 3D-printed FDTs.

### 3.2. Effect of Viscosity and Extrusion Rate on Printability, Morphological, and Physicochemical Characteristics of the 3D-Printed FDTs

**Figure 4:**
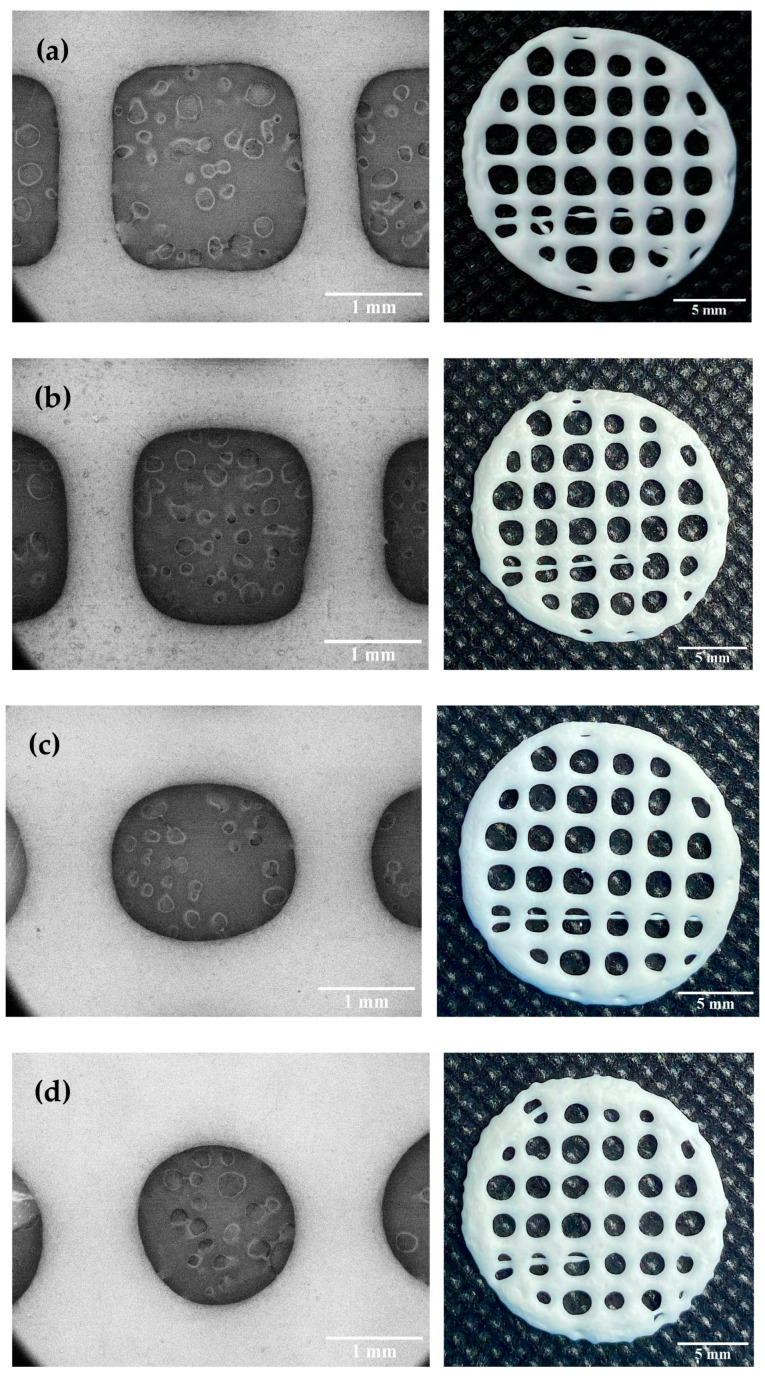
SEM images and photographs of the dried 3D-printed FDTs containing polymer:drug ratio (*w*/*w*) of 1:30 with extrusion rates of 3.5 μL/s (**a**) and 4.0 μL/s (**b**), and 1:35 with extrusion rates of 3.5 μL/s (**c**) 4.0 μL/s (**d**) and 25% of infill density.

Dimensional accuracy and shape fidelity are important factors to consider when developing 3D printed products to ensure that the 3D-printed FDTs can be reproducibly printed with acceptable appearance and contain the targeted amount of phenytoin sodium. According to our preliminary results on filament fusion analysis, 3D-printed FDTs with a 25% infill density have acceptable tablet appearance, the least merging, and the highest drug-loading content when compared with others printed with a lower or higher infill density. In this study, the results exhibited that HPMC E15 content and printing ink viscosity had a significant influence on the printing quality, dimensional accuracy, and shape fidelity of the 3D-printed FDTs. As shown in Table 2, the diameter of printing ink filament and rate of material spreading (Df_r_) were found to be significantly decreased (*p* < 0.05) as the polymer content of the printing ink increased and the ink became more viscous. Our results are in accordance with the previous studies in showing that the printing ink with a high flow behavior index (n) could spread out on the building plate after printing due to its low viscosity, leading to a larger diameter of printing ink filament than the actual nozzle size; whereas, the increase HPMC content and printing ink viscosity resulted in smaller diameter of printing ink filament, which subsequently improved the geometries’ resolution and printing quality [41,43]. Moreover, the results demonstrated that not only the HPMC E15 content (and therefore the viscosity) but also the printing parameters, such as the extrusion rate through the nozzle (22G), played an important role in the 3D-printing process and had an effect on diameter of the extruded ink filament and Df_r_. During the printing parameter optimization process, it was found that changing in extrusion rate in ranges of 3.0, 3.5, and 4.0 μL/s caused the observable changes in diameter of the extruded ink filament and pore size of the printed structures (Figure 4). The extruded filament diameter of printing ink formulation 1:30 decreased significantly (*p* < 0.05); whereas the extruded filament diameter of printing ink formulation 1:35 decreased slightly (*p* > 0.05) with a 0.5 μL/s decrease in extrusion rate. The diameters of the extruded ink with a polymer:drug ratio of 1:30 and an extrusion rate of 3.5 μL/s were found to be mostly close to the actual printing nozzle diameter (0.42 ± 0.02 mm), indicating that it is the optimum formulation that can maintain the geometric characteristics during printing. At an extrusion rate less than 3.5 μL/s, neither formulation was capable of printing FDTs with acceptable shape fidelity. When all other parameters were kept constant, too low extrusion rate (3.0 μL/s) caused flow instabilities and discontinuous printed filaments, as well as facilitating the solvent evaporation around the nozzle tip, thus leading to nozzle blockage issues.

As shown in Figure 4, all 3D-printed FDTs after drying were rounded in shape, white in color and had a porous grid structure. The SEM images of the FDTs demonstrated the printed filaments are uniform in diameter with smooth surfaces. The dimensional accuracy of the 3D-printed FDTs with a polymer:drug ratio (*w*/*w*) of 1:30 and an extrusion rate of 3.5 μL/s showed better pore size and geometry than other printing formulations. As indicated in Table 2, the printing ink of this formulation was found to spread out during the drying process in the rate of 15.65 ± 5.58% and the rate was increased to 25.69 ± 9.39% for a same formulation printed at an extrusion rate of 4.0 μL/s. The pore sizes of 3D-printed FDTs were found to decrease slightly during the drying process, resulting in a reduction in printing quality of the 3D-printed FDTs after drying. In addition, it was observed that the rate of material spreading (diffusion rate) was significantly increased to 40.07 ± 9.89 and 47.05 ± 7.79% for 3D-printed FDTs with a polymer:drug ratio of 1:35 and an extrusion rate of 3.5 and 4.0 μL/s, respectively. When the HPMC E15 content and viscosity were decreased, the actual pore shape of the 3D-printed FDTs of these two formulations was observed to be more rounded than the regular square shape which was predesigned in the CAD model. As a result, the findings of this study indicated that the actual pore shape and size of the 3D-printed FDTs were mainly influenced by the viscosity of the printing inks. The printing ink should have a high enough viscosity that allows the printing structures to stack up into three layers and keep their shape after printing and drying. Our findings are also in an agreement with the literature which suggested that the shape retention ability of the printed structure was improved by increasing the HPMC concentration [44] and the actual pore area of the printed structure became smaller than the designed pore area, resulting in a reduction in shape fidelity and printing resolution [45]. Nonetheless, these results revealed that changes in printing ink viscosity and extrusion rate had no significant effect on the peripheral dimensions or shape fidelity (SFF) of 3D-printed FDTs. The SFF of the 3D-printed FDTs is 0.92 (the printed construct dimensions comparing to original CAD design) which is a result of the merging and shrinkage of the FDTs after drying.

Furthermore, the effect of varying the viscosity and extrusion rate on the weight and thickness of 3D-printed FDTs was investigated in this study in order to optimize printing parameters and ensure the consistency and accuracy of the printing process. The weight and thickness of all 3D-printed FDTs were illustrated in Table 3. The average weight of 3D-printed FDTs with varying viscosities and extrusion rates was ranged from 0.128 to 0.164 g. As expected, the extrusion rate was found to be a very effective factor in controlling the weight of 3D-printed FDTs. As the extrusion rate increased, the weight of 3D-printed FDTs increased significantly (*p* < 0.05) and thereby may lead to inaccurate dose of phenytoin sodium. However, the narrow standard deviation (SD) of the 3D-printed FDTs weights was obtained in all formulations, implying that the fabrication of the 3D constructs using semisolid extrusion in our study is highly reproducible. This finding is consistent with previous studies that reported that 3D printing technology could offer an advantage in terms of printing precision over the traditional methods, as well as low weight variation of 3D-printed drug delivery systems that meet pharmacopeial specifications [46]. On the other hand, for the thickness uniformity of the 3D-printed FDTs, it was observed that changes in viscosity and extrusion rate had no effect on the thickness. The average thickness of all 3D-printed FDTs was similar (*p* > 0.05) across all 4 tested 3D-printed FDTs but significantly decreased (*p* < 0.05) when compared with the thickness of the CAD model (1 mm). These decreases are a result of water loss due to evaporation. The evaporation of solvent from 3D constructs could increase the rigidity of printing inks and induce shape shrinkage of printing filaments and 3D constructs during the conversion from semisolid to solid state after complete drying [47,48]. In addition, due to the high ink fluidity, the 3D constructs may have collapsed under their own weight during the drying process [49]. 

### 3.3. In Vitro Disintegration Performance of 3D-Printed FDTs

As shown in Figure S1 and Table 4, for the in vitro disintegration test, 3D-printed FDTs with a polymer:drug ratio of 1:30 and extrusion rates of 3.5 and 4.0 μL/s had a shorter disintegration time (0.81 ± 0.14 and 1.01 ± 0.01 min, respectively) than 3D-printed FDTs with a polymer:drug ratio of 1:35 and extrusion rates of 3.5 and 4.0 μL/s (1.11 ± 0.05 and 1.23 ± 0.11 min, respectively). The faster disintegration of formulation 1:30 may be attributed to their larger pore sizes and lower material spreading rate of 3D-printed FDTs, as well as their lower weight. These results are in good agreement with the data presented in Section 3.2, in which 3D-printed FDTs with a polymer:drug ratio of 1:30 and extrusion rates of 3.5 μL/s demonstrated superior geometry and larger pore size when compared with others, which was advantageous to the disintegration of the 3D-printed FDTs. The larger pore diameter may allow for the faster water uptake, thus facilitating the rapid and strong swelling characteristics of SSG and resulting in faster 3D structure disintegration. Conversely, the reduced porosity (rounded pore shape with smaller diameter) in formulation 1:35 resulted in a longer water penetrating time into the 3D-printed FDTs [50,51]. However, this study showed the promising results that all 3D-printed FDTs had an average disintegration time of less than 180 s (3 min), achieving the European Pharmacopeia (Ph.Eur.) specifications for orodispersible tablet disintegration tests [52].

### 3.4. Phenytoin Sodium Content Uniformity in 3D-Printed FDTs

In this study, the targeted content of phenytoin sodium in all 3D-printed FDTs was 75 mg. As shown in Table 4, the loading contents of phenytoin sodium in all developed 3D-printed FDTs were found to be 102.0 ± 3.6, 123.8 ± 8.4, 146.5 ± 8.2, and 167.2 ± 9.5%, respectively. The findings of this study showed that 3D-printed FDTs with a polymer:drug ratio of 1:30 and extrusion rates of 3.5 μL/s had drug content within an acceptable range of 95.0–105.0%, as endorsed by the USP [35], and range of 98.0–102.0%, as endorsed by the Ph.Eur. [52]; meanwhile, the drug content of the other three 3D-printed FDTs was found to be outside the pharmacopeia range. This could be due to the fact that when the extrusion rate increased or the printing ink viscosity decreased, the printing ink could be extruded more than its actual volume, thus leading to higher drug content in these three formulations. These results are consistent with the printability results presented in Section 3.2, which showed that the width of the printed filament with a polymer:drug ratio of 1:30 and an extrusion rate of 3.5 μL/s was similar to the nozzle diameter, allowing for the printing of an accurate dose of 3D-printed FDTs. Furthermore, this study confirmed that the optimal parameters for printing 3D-FDTs matching the designed geometry and offering the fastest disintegration time and accurate drug dosing were 0.41 mm nozzle diameter, 3.5 μL/s extrusion rate, and 10 mm/s nozzle speed. As a result, 3D-printed FDTs with a polymer:drug ratio of 1:30 and extrusion rates of 3.5 μL/s were chosen for further evaluation for their mechanical properties, in vitro release profiles, IDDSI flow characteristics and suitability for use in patients experiencing swallowing difficulties.

### 3.5. Mechanical Properties of 3D-Printed FDTs

The mechanical properties (hardness and tensile strength) of the selected 3D-printed FDT formulation (3D-printed FDTs with a polymer:drug ratio of 1:30 and extrusion rates of 3.5 µL/s) were investigated in order to assess the post-manufacturing handling capability and packaging requirements. However, there is no official guidance for determining the mechanical properties and limit hardness specification of the 3D-printed FDT reported in the pharmacopeia. In this study, the 3D-printed FDT with a polymer:drug ratio of 1:30 and extrusion rates of 3.5 µL/s had a low hardness value of 1.87 ± 0.24 N and low tensile strength of 0.69 ± 0.11 N/mm^2^. The low hardness and tensile strength of the 3D-printed FDT may be advantageous for the fast-disintegrating formulation, particularly in terms of promoting its fast disintegration [13,53]. However, special packaging is required to protect the tablets from damage prior to practical use and to improve handling safety for healthcare professionals or patients to handle them with ease in hospital settings, pharmacy settings, or at home.

### 3.6. In Vitro Release of Phenytoin Sodium

**Figure 5:**
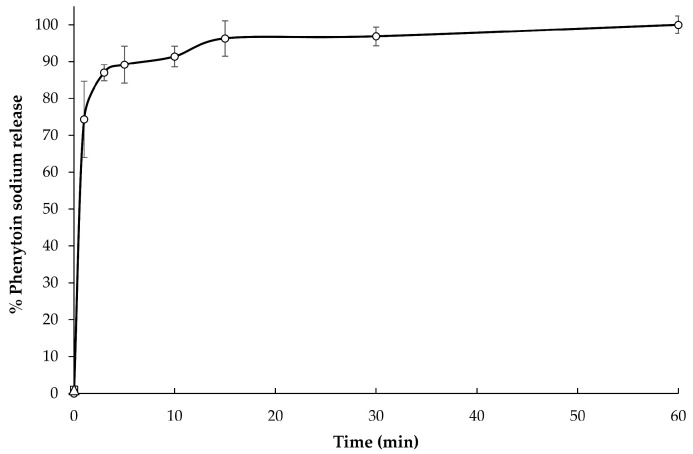
In vitro phenytoin sodium release in Tris with 1% *w*/*v* SLS buffer solution (pH 7.5).

The in vitro release profile (Figure 5) of the selected formulation (3D-printed FDTs with a polymer:drug ratio of 1:30 and extrusion rates of 3.5 µL/s) in Tris with 1% *w*/*v* SLS buffer solution (pH 7.5), which is simulated small intestinal fluid, is presented as a relationship plot between the cumulative percentage of phenytoin sodium release and time. The selected formulation exhibited rapid release behavior with an initial burst release of up to 75% of the drugs in the first 1 min of the experiment, followed by a slow constant release rate to complete drug release (100%) in 60 min. The initial burst release of phenytoin sodium in the first 1 min might be attributed to the presence of drug dissolved in water after disintegration in syringe and weak bonding of drug molecules and polymer molecules.

Furthermore, in the present study, the in vitro drug release data from the sample suspension were subjected to evaluate kinetically using various kinetic models, such as zero-order, first-order, Higuchi matrix, and Korsmeyer–Peppas models. As shown in Table 5, the Korsmeyer–Peppas model was found to be the best-fit model, with the highest correlation coefficient (*r*^2^) of 0.997 and an n value of 0.09. An n value less than 0.45 of this formulation indicated that the drug release mechanism is similar to Fickian diffusion-controlled release [54].

### 3.7. IDDSI Flow Test Results

**Figure 6:**
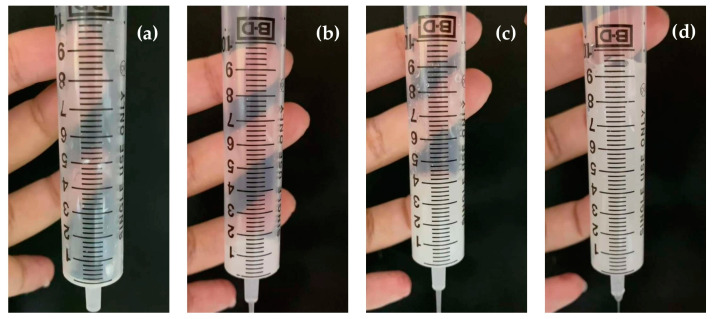
Representative images during IDDSI flow test of 3D-printed FDTs disintegrated in (**a**) water, (**b**) water mixed with thickening agents at 0.5% *w*/*v*, (**c**) water mixed with thickening agents at 1.0% *w*/*v*, and (**d**) water mixed with thickening agents at 2.0% *w*/*v*.

The results of the IDDSI flow test evaluation are displayed in Table 6 and Figure 6. After disintegrating in 10 mL of water, the liquid samples of selected 3D-printed FDTs formulation (polymer:drug ratio of 1:30 and extrusion rates of 3.5 μL/s) were evaluated for their IDDSI flow characteristics through a syringe. The results showed that there was no liquid left in the syringe after 10 s, corresponding to the IDDSI flow test level 0 (thin). It implied that the liquid sample of selected 3D-printed FDTs formulation behaves and flows like water. Despite the fact that this type of liquid sample is suitable for drinking through any type of teat/nipple, cup, or straw, as appropriate for age and skills, there are still concerns about the increased risk of aspiration and pneumonia when consumed by dysphagic patients [11,55].

For an additional recommendation to improve swallowing safety, we would suggest the option of disintegrating and dissolving the 3D-printed FDTs in water mixed with thickening agents. In this study, we also performed the IDDSI flow tests by using the water mixed with commercial thickening agent (Resource^®^ ThickenUp™ Clear, Nestlé Health Science (Deutschland) GmbH, Osthofen, Germany), which consisted of 66% of maltodextrin, 33% of xanthan gum (INS 415), and 0.6% of potassium chloride (INS 508), at 0.5%, 1.0%, and 2.0% *w*/*v*. The results showed that 3 liquid samples of 3D-printed FDTs disintegrated in water mixed with commercial thickening agent at 0.5, 1.0, and 2.0% *w*/*v* were classified as IDDSI levels 1 (slightly thick), 2 (mildly thick), and 3 (moderately thick) as the average volume of liquid remaining into the syringe after 10 s was 1.0 ± 0.2, 4.1 ± 0.1, and 9.4 ± 0.2 mL, respectively. The addition of thickening agents may make them more suitable for dysphagic patients and patients with poor tongue control.

Additionally, in order to ensure the safety and efficacy of the 3D-printed products, the robust real-time monitoring and quality process control of the fabrication of on-demand dosage forms by using semisolid 3D printing on a small scale at—or close to—the point of care, such as the use of non-destructive characterization methods and process analytical technologies (PAT), need to be taken into account in the further study. Moreover, the quality control tests, such as drug content uniformity, drug performance, and printing accuracy, must be strictly controlled in small-scale settings.

## 4. Conclusions

In this study, semisolid extrusion 3D printing was used to manufacture fast-disintegrating tablets that were filled in syringes. We note that by exploiting this technique, we may be able to achieve more accurate and precise drug dosing of narrow therapeutic index formulations in a shorter manufacturing time. This research could pave the way for point-of-care fabrication and decentralized on-site manufacturing of personalized medicines in community pharmacies and hospital settings in the near future. Notably, the effect of printing ink viscosity and extrusion rate on the printability and physicochemical properties of 3D-printed FDTs was also observed. The phenytoin-sodium-loaded, 3D-printed FDT with a polymer:drug ratio of 1:30, printed with an extrusion rate of 3.5 μL/s and a nozzle speed of 10 mm/s, was determined to be the optimal option of all of the developed 3D-printed FDT formulations, as it exhibited the least structural deformation, fastest disintegration time of less than 1 min, and most accurate drug dosing of 75 mg. To best of our knowledge, our study was the first to introduce the concept of ‘tablet-in-syringe’, in which the fast-disintegrating drug delivery system can be directly mixed with water and with the potential of a new way to accurately dose patients with dysphagia via the oral route. However, the findings of IDDSI flow test reported here suggest that the liquid sample of 3D-printed FDTs after disintegration is too thin, which may increase the choking risk when given to patients with swallowing difficulties. Thus, further development may be required to minimize this risk and to ensure that dysphagic patients can use this 3D-printed drug delivery system with ease. 

## Figures and Tables

**Table 1:** Viscosity at initial shear rate, flow behavior index (n), consistency coefficient (K), and correlation coefficient (R^2^) of the printing inks at polymer:drug ratio (*w*/*w*) of 1:25, 1:30, and 1:35.

Printing Ink Formulation	Viscosity (Pa·s ± SD)	Flow Behavior Index (n)	Consistency Coefficient (K)	Correlation Coefficient (R^2^)
1:25	961.47 ± 81.25	0.00	3316.37	0.9972
1:30	493.10 ± 7.17	0.06	1621.95	0.9968
1:35	270.25 ± 13.58	0.19	744.02	0.9931

**Table 2:** Printing quality, dimensional accuracy, and shape fidelity analysis of 3D-printed FDTs.

Printing Ink Formulation	Extrusion Rate (μL/s)	Diameter of Printing Ink Filament (mm ± SD)	Rate of Material Spreading or Df_r_ (% ± SD)	Shape Fidelity Factor or SFF
1:30	3.0	NA	NA	NA
	3.5	0.42 ± 0.02 ^a^	15.65 ± 5.58 ^a^	0.92 ± 0.01 ^a^
	4.0	0.48 ± 0.03 ^b^	25.69 ± 9.39 ^a^	0.92 ± 0.03 ^a^
1:35	3.0	NA	NA	NA
	3.5	0.78 ± 0.04 ^c^	40.07 ± 9.89 ^b^	0.92 ± 0.02 ^a^
	4.0	0.82 ± 0.01 ^c^	47.05 ± 7.79 ^b^	0.94 ± 0.01 ^a^

Note: NA (not applicable) means the printing formulations could not extrude through the nozzle or be continuously printed. For each test, means with the same letter are not significantly different. Thus, means with the different letter, e.g., ‘a’ or ‘b’ or ‘c’ are statistically different (*p* < 0.05).

**Table 3:** Weight and thickness of the 3D-printed FDTs.

Formulation	Extrusion Rate (μL/s)	Weight (g ± SD)	Thickness (mm ± SD)
1:30	3.5	0.128 ± 0.008 ^a^	0.900 ± 0.065 ^a^
	4.0	0.140 ± 0.009 ^b^	0.903 ± 0.079 ^a^
1:35	3.5	0.150 ± 0.009 ^c^	0.913 ± 0.079 ^a^
	4.0	0.164 ± 0.006 ^d^	0.934 ± 0.090 ^a^

For each test, means with the same letter are not significantly different. Thus, means with the different letter, e.g., ‘a’ or ‘b’ or ‘c’ or ‘d’ are statistically different (*p* < 0.05).

**Table 4:** In vitro disintegration time and phenytoin sodium content of the 3D-printed FDTs.

Formulation	Extrusion Rate (μL/s)	Disintegration Time (min ± SD)	Drug Content (% ± SD)
1:30	3.5	0.81 ± 0.14 ^a^	102.0 ± 3.6 ^a^
	4.0	1.01 ± 0.01 ^b^	123.8 ± 8.4 ^b^
1:35	3.5	1.11 ± 0.05 ^b^	146.5 ± 8.2 ^c^
	4.0	1.23 ± 0.11 ^b^	167.2 ± 9.5 ^c^

For each test, means with the same letter are not significantly different. Thus, means with the different letter, e.g., ‘a’ or ‘b’ or ‘c’ are statistically different (*p* < 0.05).

**Table 5:** Release kinetic data of the sample suspension containing phenytoin sodium.

Release Kinetic Model	Parameters	
zero-order	$r^{2}$	0.737
	$k_{0}\;\left(\min^{-1}\right)$	1.24
first-order	$r^{2}$	0.704
	$k_{1}\;\left(\min^{-1}\right)$	0.01
Higuchi matrix	$r^{2}$	0.898
	$k_{H}\;\left(\min^{1/2}\right)$	31.96
Korsmeyer–Peppas	$r^{2}$	0.997
	$k\;\left(\min^{-n}\right)$	76.54
	n	0.09

**Table 6:** IDDSI flow test of the 3D-printed FDTs.

Solvent	Thickening Agent Concentration (% *w*/*v*)	Volume Remaining in the Syringe after 10 s (mL ± SD)	IDDSI Level
water	-	0.0	0
water mixed with thickening agent	0.5	1.0 ± 0.2	1
	1.0	4.1 ± 0.1	2
	2.0	9.4 ± 0.2	3

## Supplementary Materials

The following supporting information can be downloaded at: https://www.mdpi.com/article/10.3390/pharmaceutics14020443/s1, Figure S1: Disintegrating behavior of 3D-printed FDT with polymer:drug ratio (*w*/*w*) of 1:30 and extrusion rates of 3.5 μL/s in syringe at different time interval 15 s (a), 30 s (b), 45 s (c), 60 s (d).
