# Supplementary material for: ‘Tablet-in-Syringe’: A Novel Dosing Mechanism for Dysphagic Patients Containing Fast-Disintegrating Tablets Fabricated Using Semisolid Extrusion 3D Printing
Source: Pharmaceutics. 2022 Feb 18;14(2):443. doi: 10.3390/pharmaceutics14020443 (PMC8879151; doi:10.3390/pharmaceutics14020443)
Supplement: Supplementary file 1 [file pharmaceutics-14-00443-s001.zip › pharmaceutics-1587238-supplementary.pdf]

pharmaceutics

Supplementary Materials: 'Tablet-in-Syringe': A Novel Dosing Mechanism for Dysphagic Patients Containing Fast-Disintegrating Tablets Fabricated Using Semisolid Extrusion 3D Printing

Pattaraporn Panraksa, Bin Zhang, Pornchai Rachtanapun, Kittisak Jantanasakulwong, Sheng Qi and Pensak Jantrawut

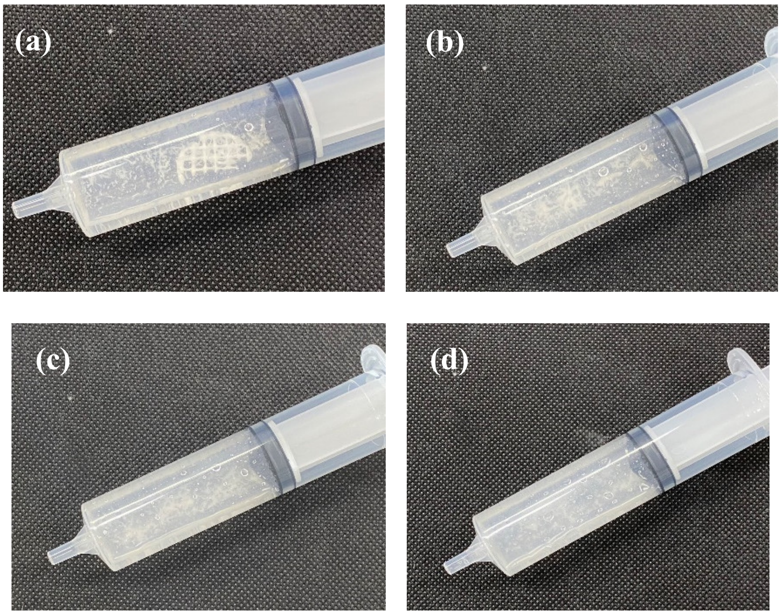

Figure S1. Disintegrating behavior of 3D-printed FDT with polymer:drug ratio (*w*/*w*) of 1:30 and extrusion rates of 3.5 μL/s in syringe at different time interval 15 s (**a**), 30 s (**b**), 45 s (**c**), 60 s (**d**).
